# Integrative Medicine for Postoperative Patients: A Survey of Korean Medicine Doctors

**DOI:** 10.1155/2017/4650343

**Published:** 2017-07-31

**Authors:** Sun-Young Moon, Kyung-Min Shin, Jae-Young Shin, O-Jin Kwon, Jun-Hwan Lee

**Affiliations:** ^1^Clinical Research Division, Korea Institute of Oriental Medicine, Daejeon 34054, Republic of Korea; ^2^Korean Medicine Life Science, University of Science and Technology (UST), Campus of Korea Institute of Oriental Medicine, Daejeon 34054, Republic of Korea

## Abstract

The purpose of this survey was to document the experience of Korean Medicine doctors (KMD) who provided postoperative care to patients through integrative medicine and to understand their opinions about integrative medicine utilization. Three researchers (two with a KMD license) of the Korea Institute of Oriental Medicine conducted the survey. The questionnaire was distributed via e-mail to the 17,041 members of the Association of Korean Medicine in 2015. In total, 487 KMD answered the questionnaires. The majority of respondents worked in a Korean Medicine (KM) clinic, KM hospital, or long-term care hospitals (94.7%). The respondents mostly treated patients after musculoskeletal (26.7%), spinal (23.7%), or neuropathic surgery (22.2%). Patients predominantly experienced pain (23.0%), fatigue and tiredness (17.4%), delayed scar recovery (13.7%), and paralysis (13.0%). We analyzed subgroups in accordance with institution of employment, specialization, and clinical experience. Most KMD wanted to utilize integrative medicine for postoperative care of patients (92.6%). Moreover, a relatively active collaboration was noted in long-term care hospitals (mean rate: 60.73% [95% CI: 42.25 to 79.20]). Further studies and clinical trials are needed to determine whether integrative medicine is essential for providing postoperative care to Korean patients.

## 1. Introduction

Integrative medicine is a collaborative approach to patient care that involves “bringing conventional and complementary approaches together in a coordinated way” and suggests a patient-centered treatment [1]. Complementary approaches mainly indicate complementary and alternative medicine, which encompass “a group of diverse medical and healthcare interventions that are not generally included under conventional medicine [2].”

In Korea, Korean Medicine (KM) plays a pivotal role in complementary therapies. KM involves the same clinical techniques as East Asian traditional medicine, such as traditional Chinese medicine (TCM). However, it also has distinct characteristics, including Sasang typology, Sa-am acupuncture, Chuna therapy, and pharmacopuncture. In recent times, KM has adapted techniques of western medicine for evaluating the efficacy of KM by known biomarkers, in order to secure the safety of KM and to validate the efficacy of KM scientifically [3].

Integrative medicine has been adapted to treat chronic diseases and postoperative adverse effects in Korea. A Stroke and Neurological Disorders Center in a KM hospital has conducted integrative stroke care, in which KM and physical medicine, involving rehabilitation, are used after neurosurgery. The number of inpatients with stroke treated using such integrative medicine has incrementally increased [4]. In addition, the KM hospitals particularly provide care for patients with musculoskeletal disorders in Korea and several US hospitals use integrative medicine for the treatment of patients with spinal disorders [5]. Clinical trials have also been conducted using different methodologies including the protocol for a prospective observational study using integrative medicine in children with cerebral palsy, protocol of three-armed randomized controlled trial for symptomatic lumbar spinal stenosis, and case series of prescribing herbal medicine and gefitinib for non-small-cell lung cancer patients [6–8].

Several surveys have been conducted on the use of treatments employing integrative medicine [5, 9, 10]; however, there has been no survey on the use of integrative medicine for postoperative care among patients in Korea. The present survey aimed to discern the methods for treating postoperative symptoms on the basis of KM and to examine the experiences and insights of KMD on the use of integrative medicine to treat patients with these symptoms.

## 2. Materials and Methods

### 2.1. Subjects

All KMD who signed up to the website of the Association of Korean Medicine (AKOM) were the subjects of this research. The survey was distributed to these KMD via e-mail on October 13, 2015, and again on October 20, 2015. A mobile phone text message, encouraging KMD to complete the survey, was sent to AKOM members simultaneously with the second survey e-mail. The survey acceptance time was 15 days.

### 2.2. Contents of Survey

Three researchers of the Korea Institution of Oriental Medicine (KIOM) developed the survey protocol and questions. Two of the members were certified KMD.

The questionnaire comprised 31 items, including specific questions, and was based on a Likert five-point scale. The survey topic of integrative medicine was divided into 3 parts. The first part asked the respondent's basic information (sex, age, type, and location of workplace, years of clinical practice experience, certification as a KMD, educational background, highest qualification, number of patients per week, and dual-certification as conventional medicine doctor and KMD).

The second part included general questions about integrative medicine. In this survey, integrative medicine in Korea was defined based on 3 concepts proposed by the Ministry of Health and Welfare in Korea (MOHW) and interorganizational collaboration in Canada [11, 12]. The first concept, latent collaboration, represents a less formal form of association between western medicine and KM and involves the use of routine common medications. The second concept entails developing collaboration between western medicine and KM, where the respective specialties are retained while working synergistically to achieve treatment efficacy. The third is active collaboration and involves optimizing the strengths of Korean and western medicine while minimizing the weaknesses.

Consequently, each of these methods could be compounded to become a new treatment approach. To summarize, this part of the survey determined KMD's current integrative medicine intervention status and monitored their requirements with respect to integrative medicine. There were seven questions in this part of the survey (necessity of integrative medicine, current status of integrative medicine usage, the rate of integrative medicine implementation, diseases frequently treated using integrative medicine, patients' visiting style, and the ideal style of integrative medicine).

Finally, the third part of the survey involved questions about the postoperative use of integrative medicine. The surgeries for which postoperative care was most often provided by KMD were ranked, and subquestions were included about integrative medicine (treatment frequency and types of acupuncture, formulations of herbal medicine and its coverage of health insurance, and additional therapies, such as moxibustion and cupping) and were added.

The full survey text can be accessed in Appendix 1 in Supplementary Material available online at https://doi.org/10.1155/2017/4650343.

### 2.3. Statistical Methods

A statistician analyzed the frequencies and used the *t*-test to compare data, by implementing SPSS version 20 (IBM Corp., Armonk, NY). A *P* value of <0.05 was considered for statistical significance.

### 2.4. Ethical Approval

This survey was approved by the institutional review board of KIOM in September 2015 (I-1509/005-002).

## 3. Results

### 3.1. Clinical Demographics

The number of certified KMD in 2014 was 22,074 [13]. A total of 17,041 members, subscribed in the website of AKOM, were approached to complete the survey, and 487 responded; that is, the response rate was 2.86%. The respondents' sex ratio was convergent with that in the KMD population and the composition of each working institute where respondents worked was equivalent to that of the survey demographics [13]. Respondents' mean age was 41.80 years, and the proportion of male KMD was 4 times greater than that of female KMD. A KM clinic was the most common place of employment (Table 1).

### 3.2. General Integrative Medicine Utilization and Needs

Approximately 90% of respondents answered that integrative medicine should be used to expand the usage of KM. However, only 34% of KMD had experience in utilizing integrative medicine. A total of 79.3% of KMD working in a KM hospital were treating patients using integrative medicine. In contrast, only 19% of KMD working in a KM clinic utilized integrative medicine. The latent collaboration was the most common type of integrative medicine used by KMD (70.8%). Only 8.4% of participants used the active collaboration type of integrative medicine.

In this survey, approximately 33% of respondents' patients visited a KM clinic or hospital by themselves after they had received treatments in conventional medicine institutions. Moreover, 32% of respondents' patients visited a KM clinic as first recourse. The most prevalent integrative medicine type was latent collaboration, based on patients' request (38%), while active collaboration was marginally attempted (1.8%). In contrast to a real-world setting, more than a half of the respondents (56%) wanted to treat patients using a developing collaboration approach, that is, in cooperation with doctors, after requesting such cooperation. In total, 24.6% of KMD respondents asserted that a conventional doctor and KMD should work together from the beginning. Integrative medicine is most commonly used in musculoskeletal and cardiovascular disorder management (27.2%). (Table 2).

### 3.3. Integrative Medicine for Postoperative Care

More than 80% of participants who were experienced in using integrative medicine had utilized integrative medicine for postoperative care (81.33%). A total of 135 respondents gave multiple responses to this question, by frequency. The highest frequency of integrative medicine utilization to care postoperative symptoms was for fracture and joint surgery (26.7%), while spinal surgery was ranked first when 135 participants graded their experience frequency for postoperative treatment (ranked first: spinal surgery = 31.1%, fracture and joint surgery = 29.6%, and brain and neuropathy surgery = 28.9%). Spinal surgery was the second most common intervention for which KMD adopted an integrative medicine approach to surgery patients. The third most frequent use of integrative medicine to relieve adverse effects of surgery intervention was for neuropathy and brain disease surgery (22.2%). The most common postoperative adverse effects treated by integrative medicine were pain, paralysis with numbness and dizziness (controlled chiefly by acupuncture), fatigue and tiredness, and delayed scar recovery (treated with herbal medicine; Table 3).

### 3.4. Classification of Respondents by Place of Employment

The proportion of KMD working in a KM hospital, clinic, or long-term care hospital was 95%. In this survey, the proportion of KMD specifically working in a KM clinic was 71%. Additionally, 80% of members of KM clinics were nonspecialist KMD, while more than 70% of KMD working in KM hospitals and long-term care hospitals were certified as KM specialists.

Respondents from KM hospitals emphasized that integrative medicine is essential for postoperative care (95.4%). Accordingly, KMD in KM hospitals utilized integrative medicine more actively than did those from other institutions (KM hospitals: 79.3%, long-term care hospitals: 69.2%, and KM clinics: 19.0%).

All 3 groups responded that latent collaboration is the most frequent type of integrative medicine implemented. The rate of collaboration by KMD in KM clinics was markedly lower than that in the other 2 groups (KM clinics: 12.4%, KM hospitals: 63.2%, and long-term care hospitals: 61.5%).

The mean frequency of integrative medicine was the highest in long-term care hospitals, that is, 60.73% (95% CI: 42.25 to 79.20). The mean rate of KM hospital was 29.76% (95% CI: 23.46 to 36.05), and that of the KM clinic was the lowest (mean rate: 6.57%, 95% CI: 4.64 to 8.51).

All KMD groups typically treated musculoskeletal, cardiovascular, and digestive diseases. Long-term care hospital KMD cared for patients who had various symptoms. They additionally used integrative medicine in digestive disorders (46.2%, KM hospital: 12.6%, KM clinic: 6.9%), musculoskeletal disorders (61.5%, KM hospital: 47.1%, KM clinic: 13.2%), and neurological disorders (38.5%, KM hospital: 24.1%, KM clinic: 13.2%) (Table 4).

The pattern of patients' hospital visits was determined based on multiple responses. In case of long-term care hospitals, patients mostly visited the hospital by themselves after visiting conventional hospitals (94%), but, in KM clinics and hospitals, patients visited the institution as first recourse (98.6% and 100%, resp.). All 3 institutional KMD answered that the ideal type of integrative medicine should be treated after joint discussion. Respondents in KM hospitals and long-term care hospitals usually asked for treatment from doctors in the same institution (KM hospital: 72.5%, *n* = 50, long-term care hospital: 94.4%, *n* = 17), while KMD in clinics were generally requested from another conventional clinic (*n* = 55, 79.7%).

Current integrative medicine execution was different. In KM clinics, KMD usually treated patients on the basis of the patients' own opinions (14.4%). In contrast, KM hospital workers treated patients by consultation with conventional medicine doctors, with no prior discussion (35.6%). In long-term care hospitals, KMD worked together with conventional doctors to perform integrative medicine (34.6%).

KM hospital members commonly had more experience in using integrative medicine for postoperative patients than did those working at long-term care hospitals and KM clinics (KM hospitals: 65.5%, long-term care hospitals: 50%, and KM clinics: 15.5%). Commonly performed surgeries were those for bones and fracture, spine, and brain and neuropathies. Patients who underwent brain surgery had been commonly treated in long-term care hospitals (92.9%). Highly treated postoperative patients had similar illnesses, regardless of the institutions involved. KMD in all 3 institutions treated for pain, numbness and paralysis, dizziness, and delay in scar recovery.

The interventions used to treat disease most frequently were acupuncture and herbal medicine (acupuncture—KM clinic: 6.3%, KM hospital: 24.1%, long-term care hospital: 42.3%, Herbal medicine—KM clinic: 4.0%, KM hospital: 19.5%, and long-term care hospital: 11.5%). KMD from all institutions usually used nonbenefit decoctions (the health insurance does not cover the costs of drugs).

### 3.5. Comparison of KMD Specialists and Nonspecialists

In this survey, more than 30% of the respondents were KMD specialists. Answers of specialists were somewhat different from those of nonspecialists. The specialists' mean age was 39.47 years, which was 3.5 years younger than that of nonspecialists (*P* value < 0.05). There were 157 KMD specialists in this survey.

KMD specialists tended to answer that integrative medicine is essential to KMD more than did nonspecialists. The proportion of KMD specialists utilizing integrative medicine (51.6%) was twice that of nonspecialists (25.8%). The KMD specialist group significantly more frequently implemented integrative medicine (19.1%) than did the nonspecialists (12.3%; *P* value = 0.011).

Specialists provided care for patients with musculoskeletal diseases more often than nonspecialists did (specialists: 32.5%, nonspecialists: 17.9%), while nonspecialists treated digestive diseases more often than did specialists (nonspecialists: 11.8%, specialists: 5.7%).

There were various responses on the ideal integrative medicine. Although both groups answered that joint discussion after inquiries (developing collaboration) was the ideal style of consultation (specialists: 63.7%, nonspecialists: 52.4%), the nonspecialist group preferred joint discussion early on (active collaboration, nonspecialists: 26.1%, specialists: 21.7%).

KMD specialists had more experience in treating postoperative patients: 43.3% of KMD specialist respondents treated patients postoperatively, while only 20.3% of nonspecialists had experience in treating patients postoperatively.

### 3.6. Grouping Respondents by Clinical Experience

Based on KMD' raw response data, KMD could be divided into 3 groups based on clinical experiences (Group 1: Clinical experience of 0–10 years, Group 2: more than 10 years to 20 years, and Group 3: more than 20 years).

All 3 groups performed integrative medicine using latent, developing, and active collaboration, in sequence. Most of the respondents worked together, but without discussion among each other.

Group 1 KMD additionally treated musculoskeletal diseases (Group 1: 27.1%, Group 2: 18.5%, and Group 3: 19.0%). KMD who had less clinical experience mainly treated brain and neurological disorders (73.2%, 63.8%, and 55.6%), and spinal surgery symptoms (73.2%, 72.3%, and 59.3%). In contrast, gynecological disorders were treated by a clinically, highly experienced group (23.2%, 27.7%, and 48.1%).

## 4. Discussion

The reputation of KM had improved as a traditional medicine before the domination of Japan, which fostered the use of western medicine in Korea, and KMD had to contend with government to revive KM. Consequently, Korea adopted a dual-medical policy since 1951, after passing of the National Medicine Services Law [14]. However, doctors practicing each of these forms of medicine have conflicting views, due to different conceptions of treatment and environments. As medical doctors and KMD cared for patients independently, patients were confused by the dual care by KMD and western medicine doctors, and patients with chronic diseases were burdened with additional treatment costs [15]. The Medical Service Act was revised in 2009, and the cooperation between hospitals has become constitutional. Correspondingly, integrative medicine has been implemented in the medical field [16].

Prior surveys in KM hospitals, which explained integrative medicine and collaboration between KMD and conventional western medicine doctors, concluded that integrative medicine had two different purposes. The first aim was therapeutic intervention to relieve patients' discomfort. In a university-owned KM hospital, spine, rehabilitation, and rheumatic disorder centers conducted comparatively active collaboration with conventional western medicine doctors. Conventional western medicine doctors mainly consulted with KMD at the behest of the patients and to relieve their pain. The second purpose was for diagnosis, in which KMD consulted with western medicine doctors, as KMD are prohibited from using medical devices, such as X-rays and ultrasound imaging systems, by the Medical Service Act Article 27 in Korea. In the 3 above-mentioned centers, more than half of consultations with conventional medicine doctors were simple diagnostic requests. KMD consulted with conventional western medicine doctors more frequently than the reverse (59% and 41%, resp.) [17]. Another KM hospital investigated KMD who treated car accident patients and collaborated with conventional medicine doctors. The survey concluded that KMD mainly consulted about treatment with conventional western medicine doctors, particularly those in the field of orthopedics and laboratory medicine, in order to obtain an accurate diagnosis with medical devices (manuscript in preparation).

This survey investigated the use of integrative medicine in the postoperative care of patients in Korea. Our research questionnaire enquired about the experience of clinicians with integrative medicine, with a particular focus on the specific intervention period for general disorders, postoperative care, and adverse effects.

More than 40% of the survey respondents had completed their Ph.D. and the proportion of KM specialists had doubled in 2015 [18]. Despite the absence of data on the educational background of KMD in 2015, their responses implied that younger KMD who had longer educational backgrounds in proportion to nonspecialized KMD were more likely to respond to the survey.

Most respondents in the survey considered that integrative medicine is required to treat postoperative patients. However, the practical usage of integrative medicine to control the adverse effects of surgery in KM institutions was insufficient for KMD. Only one-third of KMD had experience to utilize integrative medicine. Specifically, integrative medicine was applied to KM clinics only briefly.

Patients mainly visited KM institutions by themselves at first, or after visiting conventional hospitals without discussion to conventional medical doctors. Collaborations with doctors were attempted after simple inquiries, rather than by joint discussion. Most KMD in the survey wanted to utilize integrative medicine to achieve effective postoperative symptom care. In addition, KMD required active collaboration. However, the application of integrative medicine in the KM field was limited in our survey analysis.

KMD who had experience in caring for patients with integrative medicine also provided postoperative care for their patients by using integrative medicine. They chiefly treated patients with musculoskeletal, cardiac, or neuropathic conditions. KMD largely controlled musculoskeletal, spine, brain, and neuropathy surgeries. Most postoperative patients experienced pain, numbness, fatigue, and delayed scar recovery. Respondents commonly utilized acupuncture, pharmacopuncture, and herbal medicine decoctions not covered by medicine insurance system to care for postoperative patients. The survey reflected frequently used KM interventions in Korea. In a survey of the use of KM by KMD in patients in 2014, by the Korea Health Industry Development Institute, the pivotal treatment methods were acupuncture (59.2%), herbal medicine decoction (27.6%), and herbal preparation (4.9%) [19].

According to the health insurance statistics from 2014, low back pain (M54.5) was the most frequent among 10 conditions in inpatients in the field of KM; 55,615 patients were diagnosed with this condition. The second most common condition was gonarthrosis (M17), and the third was dislocation, sprain, and strain of joints and ligaments of the lumbar spine and pelvis (S33). The first among 10 diseases in outpatients was also low back pain, which was diagnosed in 4,260,471 patients. The second most common disease was soft tissue disorder, unspecified (M79.9). The third condition was M17 and S33. Patients who visited KM hospitals also suffered from the above diseases. The codes for the disease diagnosis followed ICD-10 2010 version [20].

Most KMD worked in KM clinics, KM hospitals, and long-term care hospitals. In this survey, integrative medicine was predominantly practiced in KM hospitals and long-term care hospitals, which have departments both of KM and of conventional medicine. In KM clinics, cooperation between conventional doctors and KMD was less active than in the other institutions. Since the Medical Service Act related to hiring medical professionals in different institutions had been revised in 2009, cooperation in KM clinics is mainly conducted by requirement of patients, while collaboration in KM hospitals and long-term care hospitals is mainly led by medical professionals [21]. Political intervention is required to ensure active collaboration between KMD and conventional medicine doctors in KM clinics.

Cooperation in long-term care hospitals was based on an understanding of the medical history of collaboration between conventional western medicine and KM in South Korea. Long-term care hospitals are medical institutions established by medical doctors or KMD in order to treat patients over the long term, where more than 30 patients can be hospitalized concurrently, which have largely been established since 2005 [18]. The number of western medicine doctors increased from 1.3 to 2.1 per 100 beds and KMD increased from 0.1 to 0.7, during the period 2005 to 2013 [22]. The number of KMD working in long-term care hospitals was 1244 in 2014. More than 90% of KMD who worked at hospitals were working in the long-term care hospitals. In 2008, the medical insurance fee in long-term care hospitals changed to a fixed medical insurance fee per day [23].

Furthermore, collaboration between KMD and conventional medicine doctors can increase the income of long-term care hospitals by means of fee-for-service, while per diem payment is applied for hospitalization in conventional medicine departments situated in long-term care hospitals. Since long-term care hospitals mainly care for patients with chronic diseases and chronically ill patients in Korea have favored KM, collaboration can frequently be implemented [24].

In the case of KM hospitals, the institution can hire medical practitioners to make a diagnosis using methods such as computed tomography, magnetic resonance imaging, radiography, and ultrasonography, which KMD cannot use given the Medical Service Act, Article 27, in Korea, and technical needs in KM hospitals also support frequent implementation of collaboration. Consequently, integrative medicine can be implemented suitably in KM hospitals and in long-term care hospitals.

The survey assessed KMD specialist certification. The first KMD specialists were certified in 2002. According to the 2014 KM Chronology, around 11% of KMD were specialists [24]. In this survey, 20% of respondents were KMD specialists. They implemented integrative medicine in postoperative patients more often than nonspecialists, emphasizing the necessity for integrative medicine.

Respondents with more clinical experience tended to treat patients with gynecological disease more often than did the respondents with less clinical experience. KMD working in KM clinics also treated patients with gynecological disease. As the relationship between clinical experience and the frequency of treating gynecological disorders cannot be unambiguously defined by the present survey, further studies are needed.

There are several limitations associated with this survey. First, this was a cross-sectional study and used subjective indicators, such as the rate of implementation of integrative medicine in the comprehensive treatment provided by KMD. Second, the answer rate was relatively low in proportion to the number of members in AKOM, as compared to other survey results [9]. E-mail answers partly represented the respondents' real-time interventions. Consequently, the survey was not analyzed to determine a cause-and-effect relationship between other statistics, and studies such as those using the national health and insurance data.

A previous study concluded that the collaboration rate of long-term care hospitals was low in proportion to the rate of collaboration in KM hospitals, while both institutions actively introduced collaboration after the hiring of both KMD and conventional medicine doctors was authorized in both types of institutions [21]. This survey conclusion was not identical to that of a prior study, but we could not analyze the correlation of these two studies and our survey questions partly reflect the real world of integrative medicine in long-term care hospitals. Additional studies need to determine the real status and depth of collaboration in long term care hospitals and compare the strengths and shortfalls of this and previous studies in this field.

Further qualitative studies based on large datasets are required to determine the real impact of KM. Additionally, the recognition by non-KMD of the utility of integrative medicine can be improved. We believe that clinical trials and observational studies can be conducted to reveal the efficiency of integrative medicine in the postoperative care of patients.

## Supplementary Material

The questionnaire aimed to analyze the utilization and opinion of integrative medicine to postoperative care by KMD.

## Figures and Tables

**Table 1:** Demographics of 487 KMD respondents and all KMD in 2014.

	Respondents	Total KMD in 2014
Sex (%)		
Male	397 (82)	
Female	90 (19)	
Total	487 (100)	22,074 (100)
Mean age (SD, 95% CI), years	41.8 (8.7, 41.0–42.6)	
Working institute (%)		
Community health center	8 (1.6)	917 (4.2)
KM clinic	348 (71.5)	14,798 (67)
KM hospital	87 (17.9)	1446 (6.6)
Hospital	3 (0.6)	1774 (8)
General hospital	7 (1.4)	25 (0.1)
Long-term care hospital	26 (5.3)	1217 (5.5)
Institution and research center	4 (0.8)	
Others	4 (0.8)	1897 (8.6)
Total	487 (100)	22,074 (100)
Mean clinical experiences (SD), years	14.4 (7.9)	
Major (%)		
Nonspecialist	331 (68)	19,602 (79.8)
KM internal	50 (10)	919 (3.7)
Gynecology	17 (3.5)	208 (0.9)
Pediatrics	5 (1)	91 (0.4)
Ophthalmology/ENT/dermatology	12 (2.5)	140 (0.6)
Sasang	4 (0.8)	134 (0.5)
Rehabilitation	25 (5)	326 (1.3)
Psychology	5 (1)	148 (0.6)
Acupuncture	37 (7.6)	506 (2)
Others	1 (0.2)	2472 (10)
Total	487 (100)	
Final education (%)		
Bachelor's degree	189 (38.8)	
Master's degree	101 (20.7)	
Ph.D.	196 (40.3)	
Others	1 (0.2)	
Total	487 (100)	
Dual certification (%)		
Yes	4 (0.8)	247 (1.0)
No	483 (99.2)	21,827 (99.0)
Total	487 (100)	22,074 (100)
Mean number of patients seen per week (%)		
Less than 50	69 (14.2)	
50–99	90 (18.5)	
100–199	186 (38.2)	
200–299	95 (19.5)	
300–399	31 (6.3)	
400–499	7 (1.4)	
More than 500	6 (1.2)	
N/A	3 (0.6)	
Total	487 (100)	

**Table 2:** Status of integrative medicine usage by KMD (multiple responses).

Diseases applying integrative medicine (%)	Number of KMD who had experience in using integrative medicine (%)
Musculoskeletal disorders	108 (17.1)
Cardiovascular disorders	63 (10.0)
Neurological disorders	53 (8.3)
Digestive disorders	46 (7.3)
Respiratory disorders	45 (7.2)
Gynecologic disorders	43 (6.8)
Rheumatic diseases	33 (5.2)
Endocrine disorders	32 (5.0)
Mental disorders	30 (4.7)
Allergic disorders	25 (3.9)
Dermatological disorders	25 (3.9)
Urinary system disorders	24 (3.8)
Hematologic and oncologic disorders	23 (3.6)
Otorhinolaryngology disorders	21 (3.3)
Pediatric disorders	15 (2.3)
Infectious diseases	12 (1.9)
Ophthalmological disorders	11 (1.7)
Kidney diseases	10 (1.6)
Oral system disorders	5 (0.7)
Others	5 (0.7)
Total	629 (100)

**Table 3:** Frequency of highly used interventions treating postoperative symptoms.

		Acupuncture			Herbal medicine	
	Fracture and joint surgery (%)	Spine surgery	Brain and neuropathy surgery	Fracture and joint surgery	Spine surgery	Brain and neuropathy surgery
Pain	59 (53.2)	52 (51.0)	23 (28.8)	8 (6.8)	7 (8.0)	3 (2.9)
Paralysis and numbness	19 (17.1)	28 (27.5)	34 (42.5)	4 (3.4)	4 (4.5)	8 (7.6)
Delay of scar recovery	14 (12.6)	10 (9.8)	5 (6.3)	34 (29.1)	23 (26.1)	4 (3.8)
Fatigue and tiredness	3 (2.7)	3 (2.9)	N/A	30 (25.6)	34 (38.6)	35 (33.3)
Dizziness	N/A	N/A	5 (6.3)	N/A	1 (1.1)	23 (21.9)
Total	111 (100)	102 (100)	80 (100)	117 (100)	88 (100)	105 (100)

**Table 4:** Frequently treated diseases using integrative medicine in KM clinics and hospitals.

	KM clinic	KM hospital	Long-term care hospital
Musculoskeletal disorders	46 (15.0)	41 (18.8)	16 (19.3)
Cardiovascular disorders	27 (8.8)	26 (11.9)	9 (10.8)
Digestive disorders	24 (7.8)	11 (5.0)	12 (14.5)
Gynecologic disorders	21 (6.9)	15 (6.9)	3 (3.6)
Respiratory disorders	20 (6.5)	17 (7.8)	5 (6.0)
Rheumatic diseases	19 (6.2)	7 (3.2)	6 (7.2)
Mental disorders	19 (6.2)	7 (3.2)	4 (4.8)
Dermatological disorders	19 (6.2)	7 (3.2)	N/A
Neurological disorders	17 (5.6)	21 (9.6)	10 (12.0)
Allergic disorders	16 (5.2)	7 (3.2)	2 (2.4)
Endocrine disorders	15 (4.9)	13 (6.0)	3 (3.6)
Otorhinolaryngologic disorders	12 (3.9)	7 (3.2)	1 (1.2)
Ophthalmological disorders	9 (2.9)	3 (1.4)	N/A
Urinary System disorders	9 (2.9)	7 (3.2)	7 (8.4)
Hematologic and oncologic disorders	7 (2.3)	8 (3.7)	3 (3.6)
Pediatric disorders	7 (2.3)	7 (3.2)	1 (1.2)
Kidney diseases	5 (1.6)	4 (1.8)	1 (1.2)
Infectious diseases	5 (1.6)	8 (3.7)	N/A
Oral system disorders	5 (1.6)	1 (0.5)	N/A
Others	4 (1.3)	1 (0.5)	N/A
Total	306 (100)	218	83 (100)
