# Supplementary material for: Integrative Medicine for Postoperative Patients: A Survey of Korean Medicine Doctors
Source: Evid Based Complement Alternat Med. 2017 Jul 31;2017:4650343. doi: 10.1155/2017/4650343 (PMC5610882; doi:10.1155/2017/4650343)
Supplement: Supplementary file 1 — The questionnaire aimed to analyze the utilization and opinion of integrative medicine to postoperative care by KMD. [file 4650343.f1.docx]

*Appendix 1.*

**Integrative medicine for postoperative patients: a survey of Korean Medicine Doctors**

1. General Information
2. Sex

□ Male □ Female

1. Age: ___ years old
2. Working Information
3. At which institution is your current workplace located?

□ Health institution □ Korean Medicine Clinic □ Korean Medicine Hospital □ Clinic □ Hospital □ General hospital □ Dental Clinic □ Dental Hospital □ Long-term Care Hospital □ Research Institutions □ Other ________________

2) Where is your workplace?

□ Seoul Special City □ Incheon □ Daejeon □ Daegu □ Busan □ Ulsan □ Gwang-ju □ Sejong □ Gyeonggi-do □ Gangwon-do □ Gyeongsangbuk-do □ Gyeongsang-namdo □ Jeollabuk-do □ Jeollanam-do □ Chungcheongbuk-do □ Chungcheongnam-do □ Jeju-do □ Other______ □ N/A

1. How many years have you been working as a KMD? ________years
2. Are you a specialist of KMD? If you answered yes, what is your major subject?

□ Non-specialist

□ Specialist

(Major: □Internal □ Gynecology □ Pediatric □ ENT □ Sasang-topography □ Rehabilitation □ Psychology □ Acupuncture □ Other)

5) What is your highest qualification?

□ Bachelor □ Master’s degree □ Ph.D □ Others __________

6) Are you a dual-certified as both KMD and conventional medical doctor?

□ Yes □ No

7) What is the mean number of patients you see per week?

□ Less than 50 □ 50−99 □ 100−199 □ 200−299 □ 300−399 □ 400−499 □ More than 500 □ N/A

- **General questions for integrative medicine**

In this questionnaire, integrative medicine is defined as all activities that meet any of the following three criteria:

| 1. Latent collaboration: A less formal form of routine association in regard to common medications.  2. Developing collaboration: A collaboration between western medicine and Korean medicine while retaining their own specialties and complementing for improved treatment efficacy.  3. Active collaboration: Optimizing the strength of Korean medicine and western medicine and minimizing the weaknesses of both types of medicine. |
| --- |

1. Do you think that integrative medicine is essential to KMD?

□ Yes □ No □ Don’t Know □ Other __________________

2. Are you currently implementing integrative medicine?

□ Yes □ No (If you answered No, you can end this survey after you have answered the 7^th^ question.)

3. What proportion of patients have you treated using integrative medicine? ___Percent

4. What disease are you currently treating by integrative medicine? (Multiple responses are permitted.)

□ Respiratory Diseases □ Cardiac Diseases □ Digestive Diseases □ Hematological Diseases □ Endocrine Diseases □ Allergies □ Kidney diseases □ Infections □ Rheumatic Diseases □ Opthalmopathies □ ENT diseases □ Gynecological Diseases □ Pediatric Diseases □ Psychological disease □ Dermatological disease □ Musculoskeletal Disease □ Neuropathies □ Urinary Diseases □Oral System Diseases □ Others ________________

5. If you are performing integrative medicine, how do your patients attend medical care?

(Please prioritize the patients’ visiting style in terms of the first, second, and third most common manner)

□ First time visiting

□ Patients visit the Korean Medicine Clinic (or Hospital) by themselves after they attended western medicine clinics.

□ Transferred by western medicine clinic at another institution

□ Transferred by western medicine clinic at your institution (including co-working clinics and centers)

6. If you are performing integrative medicine, what style are you using?

□ Consultation based on patients’ opinion.

□ (Between KMD and doctor) co-request, without joint discussion

□ (Between KMD and doctor) co-request before treatment, with joint discussion

□ (Between KMD and doctor) negotiation with one another before joint treatment

7. What is the ideal type of integrative medicine in your opinion?

□ Co-treatment based on the patient’s opinion

□ (Between KMD and doctor) parallel treatment, without negotiation, after consultation

□ (Between KMD and doctor) joint treatment after discussion

□ (Between KMD and doctor) joint treatment after initial discussion

**◆ Question for integrative medicine care of post-operative patients**

1. Do you have experience in treating post-operative patients using integrative medicine?

□ Yes □ No (If you answered no, go to the end of the questionnaire)

2. What types of surgery have the patients you are currently treating by integrative medicine undergone? (Please prioritize the first, second, and third most frequent types of surgery)

□ Brain & Neuropathic surgery_____ □ Spinal surgery _____ □ Fracture and Joint surgery_____ □ Gynecological surgery_____ □ Plastic surgery_____ □ ENT surgery_____ □ Eye surgery_____ □ Cancer surgery_____ □ Oral surgery □ Others ____________________________

3. What are the most common symptoms the patients in Question 2 are experiencing? (Multiple responses are accepted. If you have multiple answers, please prioritize the first, second, and third most frequent symptoms.)

□ Pain_____ □ Fever and chills _____ □Nausea or Vomiting_____ □ Dizziness_____ □ Arrhythmia_____ □ Insomnia_____ □ Diarrhea_____ □ Constipation_____ □ Dyspepsia and anorexia _____ □ Itchiness_____ □ Allergy_____ □ Inflammation_____ □ Fatigue and decreased fitness_____ □ Melena, hemoptysis, hematemesis, and bleeding_____ □ Thirst_____ □ Hyperhidrosis_____ □ Alopecia_____ □ Paralysis and numbness_____ □ Depression_____ □ Cough, asthma, and dyspnea_____ □ Abdominal pain_____ □ Dysmenorrhea_____ □ Edema (Water retention)_____ □ Delay of scar recovery_____ □ Others __________________________ ______

4. What is the treatment method used for patients who have the symptoms in Question 3? (Select the most frequently used treatment.)

□ Herbal medicine (Internal medicine) □ Herbal medicine (External) / < Go to point 4-1 > □ Acupuncture (If you did not choose acupuncture or pharmacopuncture, the method should be specified in ‘other’) < Go to point 4-2 > □ Cupping □ Moxibustion □ Chuna □ Pharmacopuncture □ Other ___________________ /< Go to point 4-3 >

| 4-1. If your treatment method is herbal medicine (Internal medicine/External)  Name of drugs: ____________________________________________  Formulation: □ Decoction □ Pills □ Powders □ Other ______________________________  Medical insurance cover: □ Benefit □ Non-benefit □ Others ______________________________  Number of doses/day: □ once per day □ twice per day □ three times per day □ Other ______________________________  Duration: □ less than 3 days □ more than 3 days □ more than 5 days □ more than 7 days □ more than 10 days □ more than 15 days □ more than 30 days □ Other ______________________________ |
| --- |

| 4-2. If your treatment method is acupuncture  Acupoints (3−4 chief acupoints): ____________ ____________ ____________ ____________ ____________  Number of treatments: /day /week □ Other ______________________________  (For example, if you treat twice per day, three times per week, indicate this as 2 /day 3 /week)  Treatment period: □ less than 7 days □ more than 7 days □ more than 15 days □ more than 1 month □ more than 2 months □ more than 3 months  □ Other ______________________________ |
| --- |

| 4-3 If your treatment methods are non-pharmacological (cupping, moxibustion, Chuna, pharmacopuncture)  Number of treatments: /day /week □ Other ______________________________  (For example, if you treat twice per day, three times per week, indicate this as 2 /day 3 /week)  Treatment period: □ less than 7 days □ more than 7 days □ more than 15 days □ more than 1 month □ more than 2 months □ more than 3 months  □ Other ______________________________ |
| --- |

5. What is the treatment method used for patients who have symptoms ranked number 2 in Question 3? (You should select the most frequently used method).

□ Herbal medicine (Internal medicine) □ Herbal medicine (External) / < Go to point 5-1 > □ Acupuncture (If you did not choose acupuncture nor pharmacopuncture, the method should be written down in ‘other’) <Go to point 5-2 > □ Cupping □ Moxibustion □ Chuna □ Pharmacopuncture □ Others ___________________ / < Go to point 5-3 >

| 5-1. If your treatment method is herbal medicine (Internal medicine/External)  Name of drugs: ____________________________________________  Formulation: □ Decoction □ Pills □ Powders □ Others ______________________________  Medical insurance cover: □ Benefit □ Non-benefit □ Others ______________________________  Dose per day: □ once per day □ twice per day □ three times per day □ Other ______________________________  Duration: □ less than 3 days □ more than 3 days □ more than 5 days □ more than 7 days □ more than 10 days □ more than 15 days □ more than 30 days □ Other ______________________________ |
| --- |

| 5-2. If your treatment method is acupuncture  Acupoints (Chief acupoints 3−4): ____________ ____________ ____________ ____________ ____________  Number of treatment: /day /week □ Other ______________________________  (For example, if you treat twice per day, three times per week, indicate this as 2 /day 3 /week)  Treatment period: □ less than 7 days □ more than 7 days □ more than 15 days □ more than 1 month □ more than 2 months □ more than 3 months  □ Other ______________________________ |
| --- |

| 5-3 If your treatment methods are non-pharmacological (cupping, moxibustion, Chuna, pharmacopuncture)  Number of treatments: /day /week □ Other ______________________________  (For example, if you treat twice per day, three times per week, indicate this as 2 /day 3 /week)  Treatment period: □ less than 7 days □ more than 7 days □ more than 15 days □ more than 1 month □ more than 2 months □ more than 3 months  □ Others ______________________________ |
| --- |

6. What is the treatment method used for patients who have symptoms ranked number 3 in Question 3? (You should select the most frequently used one.)

□ Herbal medicine (Internal medicine) □ Herbal medicine (External) / < Go to point 6-1 > □ Acupuncture (If you did not choose acupuncture or pharmacopuncture, the method should be written down in ‘other’) < Go to point 6-2 > □ Cupping □ Moxibustion □ Chuna □ Pharmacopuncture □Other ___________________ / < Go to point 6-3 >

| 6-1. Your treatment method is herbal medicine (Internal medicine/External)  Name of drugs: ____________________________________________  Formulation: □ Decoction □ Pills □ Powders □ Others ______________________________  Medical insurance cover: □ Benefit □ Non-benefit □ Others ______________________________  Administration of drugs: □ once per day □ twice per day □ three times per day □ Other ______________________________  Duration: □ less than 3 days □ more than 3 days □ more than 5 days □ more than 7 days □ more than 10 days □ more than 15 days □ more than 30 days □ Others ______________________________ |
| --- |

| 6-2. If your treatment method is acupuncture  Acupoints (3−4 chief acupoints): ____________ ____________ ____________ ____________ ____________  Number of treatment: /day /week □ Other ______________________________  (For example, if you treat twice per day, three times per week, indicate this as 2 /day 3 /week)  Treatment period: □ less than 7 days □ more than 7 days □ more than 15 days □ more than 1 month □ more than 2 months □ more than 3 months  □ Others ______________________________ |
| --- |

| 6-3 If your treatment methods are non-pharmacological (cupping, moxibustion, Chuna, pharmacopuncture)  Number of treatments: /day /week □ Other ______________________________  (For example, if you treat twice per day, three times per week, indicate this as 2 /day 3 /week)  Treatment period: □ less than 7 days □ more than 7 days □ more than 15 days □ more than 1 month □ more than 2 months □ more than 3 months  □ Other ______________________________ |
| --- |

7. What is the second most common symptom that patients who underwent surgery in Question 2 experienced (Multiple responses are accepted: If you have multiple answers, please prioritize them from first to third by frequency)

□ Pain_____ □ Fever and chills _____ □ Nausea/Vomiting_____ □ Dizziness_____ □ Arrhythmia_____ □ Insomnia_____ □ Diarrhea_____ □ Constipation_____ □ Dyspepsia and anorexia_____ □ Itchiness _____ □ Allergies _____ □ Inflammation_____ □ Fatigue and decreased fitness_____ □ Melena, hemoptysis, hematemesis, and bleeding_____ □ Thirst_____ □ Hyperhidrosis_____ □ Alopecia_____ □ Paralysis and numbness_____ □ Depression_____ □ Cough, asthma, dyspnea_____ □ Abdominal pain_____ □ Dysmenorrhea_____ □ Edema (Water retention)_____ □ Delay of scar recovery_____ □ Other __________________________

8. What is the treatment method used for patients who have symptoms ranked number 1 in Question 7? (You should select the most frequently used method.)

□ Herbal medicine (Internal medicine) □ Herbal medicine (Externals) / < Go to point 8-1 > □ Acupuncture (If you did not choose acupuncture or pharmacopuncture, the method should be written down in ‘other’) < Go to point 8-2 > □ Cupping □ Moxibustion □ Chuna □ Pharmacopuncture □Other ___________________ / < Go to point 8-3 >

| 8-1. If your treatment method is herbal medicine (Internal medicine/External)  Name of drugs: ____________________________________________  Formulation: □ Decoction □ Pills □ Powders □ Other ______________________________  Medical insurance cover: □ Benefit □ Non-benefit □ Other ______________________________  Dosage per day: □ once per day □ twice per day □ three times per day □ Other ______________________________  Duration: □ less than 3 days □ more than 3 days □ more than 5 days □ more than 7 days □ more than 10 days □ more than 15 days □ more than 30 days □ Others ______________________________ |
| --- |

| 8-2. If your treatment method is Acupuncture  Acupoints (3−4 chief acupoints): ____________ ____________ ____________ ____________ ____________  Number of treatment: /day /week □ Others ______________________________  (For example, if you treat twice per day, three times per week, indicate this as 2 /day 3 /week)  Treatment period: □ less than 7 days □ more than 7 days □ more than 15 days □ more than 1 month □ more than 2 months □ more than 3 months  □ Others ______________________________ |
| --- |

| 8-3 If your treatment method is not acupuncture nor herbal medicine (cupping, moxibustion, Chuna, pharmacopuncture)  Number of treatment: /day /week □ Others ______________________________  (For example, if you treat twice per day, three times per week, indicate this as 2 /day 3 /week)  Treatment period: □ less than 7 days □ more than 7 days □ more than 15 days □ more than 1 month □ more than 2 months □ more than 3 months  □ Others ______________________________ |
| --- |

9. What is the treatment method used for patients who have symptoms ranked number 2 in Question 7? (You should select the most frequently used one.)

□ Herbal medicine (Internal medicine) □ Herbal medicine (External) / < Go to point 9-1 > □ acupuncture (If you did not choose acupuncture or pharmacopuncture, the method should be written down in ‘other’) < Go to point 9-2 > □ Cupping □ Moxibustion □ Chuna □ Pharmacopuncture □Other ___________________ / < Go to point 9-3 >

| 9-1. If your treatment method is herbal medicine (Internal medicine/External)  Name of drugs: ____________________________________________  Formulation: □ Decoction □ Pills □ Powders □ Others ______________________________  Medical insurance cover: □ Benefit □ Non-benefit □ Others ______________________________  Dose per day: □ one time per day □ two times per day □ three times per day □ Other ______________________________  Duration: □ less than 3 days □ more than 3 days □ more than 5 days □ more than 7 days □ more than 10 days □ more than 15 days □ more than 30 days □ Other ______________________________ |
| --- |

| 9-2. If your treatment method is acupuncture  Acupoints (3−4 chief acupoints): ____________ ____________ ____________ ____________ ____________  Number of treatment: /day /week □ Other ______________________________  (For example, if you treat twice per day, three times per week, indicate this as 2 /day 3 /week)  Treatment period: □ less than 7 days □ more than 7 days □ more than 15 days □ more than 1 month □ more than 2 months □ more than 3 months  □ Others ______________________________ |
| --- |

| 9-3 If your treatment method is not acupuncture nor herbal medicine (cupping, moxibustion, Chuna, pharmacopuncture)  Number of treatment: /day /week □ Other ______________________________  (For example, if you treat twice per day, three times per week, indicate this as 2 /day 3 /week)  Treatment period: □ less than 7 days □ more than 7 days □ more than 15 days □ more than 1 month □ more than 2 months □ more than 3 months  □ Other ______________________________ |
| --- |

10. What is the treatment method used for patients who have symptoms ranked number 3 in Question 7? (You should select the most frequently used one.)

□ Herbal medicine (Internal medicine) □ Herbal medicine (External) / < Go to point 10-1 > □ acupuncture (If you did not choose acupuncture or pharmacopuncture, the method should be written down in ‘other’) < Go to point 10-2 > □ Cupping □ Moxibustion □ Chuna □ pharmacopuncture □ Other _________________ / < Go to point 10-3 >

| 10-1. If your treatment method is herbal medicine (Internal medicine/External)  Name of drugs: ____________________________________________  Formulation: □ Decoction □ Pills □ Powders □ Others ______________________________  Medical insurance cover: □ Benefit □ Mon-benefit □ Others ______________________________  Dose per day: □ one time per day □ two times per day □ three times per day □ Other ______________________________  Duration: □ less than 3 days □ more than 3 days □ more than 5 days □ more than 7 days □ more than 10 days □ more than 15 days □ more than 30 days □ Other ______________________________ |
| --- |

| 10-2. If your treatment method is acupuncture  Acupoints (3−4 chief acupoints): ____________ ____________ ____________ ____________ ____________  Number of treatment: /day /week □ Other ______________________________  (For example, if you treat twice per day, three times per week, indicate this as 2 /day 3 /week)  Treatment period: □ less than 7 days □ more than 7 days □ more than 15 days □ more than 1 month □ more than 2 months □ more than 3 months  □ Other ______________________________ |
| --- |

| 10-3 If your treatment method is not acupuncture nor herbal medicine (cupping, moxibustion, Chuna, pharmacopuncture)  Number of treatment: /day /week □ Other ______________________________  (For example, if you treat two times per day, three times per week - 2 /day 3 /week)  Treatment period: □ less than 7 days □ more than 7 days □ more than 15 days □ more than 1 month □ more than 2 months □ more than 3 months  □ Others ______________________________ |
| --- |

11. What is the third most common symptom that patients who underwent surgery in Question 2 experienced (Multiple responses are accepted: If you have multiple answers, please prioritize them from first to third by frequency)

□ Pain_____ □ Fever and chills _____ □ Nausea/Vomiting_____ □ Dizziness_____ □ Arrhythmia_____ □ Insomnia_____ □ Diarrhea_____ □ Constipation_____ □ Dyspepsia and anorexia_____ □ Itchiness _____ □ Allergies _____ □ Inflammation_____ □ Fatigue and decreased fitness_____ □ Melena, hemoptysis, hematemesis, and bleeding_____ □ Thirst_____ □ Hyperhidrosis_____ □ Alopecia_____ □ Paralysis and numbness_____ □ Depression_____ □ Cough, asthma, and dyspnea_____ □ Abdominal pain_____ □ Dysmenorrhea_____ □ Edema (Water retention)_____ □ Delay of scar recovery_____ □ Others __________________________

12. What is the treatment method used for patients who have symptoms ranked number 1 in Question 11? (You should select the most frequently used method).

□ Herbal medicine (Internal medicine) □ Herbal medicine (External) /< Go to point 12-1 >□ Acupuncture (If you did not choose acupuncture or pharmacopuncture, the method should be written down in ‘other’) < Go to point 12-2 > □ Cupping □ Moxibustion □ Chuna □ Pharmacopuncture □Others _________________ / <Go to point 12-3>

| 12-1. If your treatment method is herbal medicine (Internal medicine/External)  Name of drugs: ____________________________________________  Formulation: □ Decoction □ Pills □ Powders □ Other ______________________________  Medical insurance cover: □ Benefit □ Non-benefit □ Other ______________________________  Dosage per day: □ one time per day □ two times per day □ three times per day □ Other ______________________________  Duration: □ less than 3 days □ more than 3 days □ more than 5 days □ more than 7 days □ more than 10 days □ more than 15 days □ more than 30 days □ Others ______________________________ |
| --- |

| 12-2. If your treatment method is acupuncture  Acupoints (3−4 chief acupoints): ____________ ____________ ____________ ____________ ____________  Number of treatment: /day /week □ Others ______________________________  (For example, if you treat twice per day, three times per week, indicate this as 2 /day 3 /week)  Treatment period: □ less than 7 days □ more than 7 days □ more than 15 days □ more than 1 month □ more than 2 months □ more than 3 months  □ Others ______________________________ |
| --- |

| 12-3 If your treatment method is not acupuncture nor herbal medicine (cupping, moxibustion, Chuna, pharmacopuncture)  Number of treatment: /day /week □ Other ______________________________  (For example, if you treat twice per day, three times per week, indicate this as 2 /day 3 /week)  Treatment period: □ less than 7 days □ more than 7 days □ more than 15 days □ more than 1 month □ more than 2 months □ more than 3 months  □ Others ______________________________ |
| --- |

13. What is the treatment method used for patients who have symptoms ranked number 2 in Question 11? (You should select the most frequently used method).

□Herbal medicine (Internal medicine) □Herbal medicine (External) < Go to point 13-1 > □Acupuncture (If you did not choose acupuncture or pharmacopuncture, the method should be written down in ‘other’) < Go to point 13-2 > □Cupping □Moxibustion □Chuna □ Pharmacopuncture □Other _______________________ < Go to point 13-3 >

| 13-1. If your treatment method is herbal medicine (Internal medicine/External)  Name of drugs: ____________________________________________  Formulation: □ Decoction □ Pills □ Powders □ Other ______________________________  Medical insurance cover: □ Benefit □ Non-benefit □ Other ______________________________  Dosage per day: □ one time per day □ two times per day □ three times per day □ Other ______________________________  Duration: □ less than 3 days □ more than 3 days □ more than 5 days □ more than 7 days □ more than 10 days □ more than 15 days □ more than 30 days □ Other ______________________________ |
| --- |

| 13-2. If your treatment method is acupuncture  Acupoints (3−4 chief acupoints): ____________ ____________ ____________ ____________ ____________  Number of treatment: /day /week □ Other ______________________________  (For example, if you treat twice per day, three times per week, indicate this as 2 /day 3 /week)  Treatment period: □ less than 7 days □ more than 7 days □ more than 15 days □ more than 1 month □ more than 2 months □ more than 3 months  □ Other ______________________________ |
| --- |

| 13-3 If your treatment method is not acupuncture nor herbal medicine (cupping, moxibustion, Chuna, pharmacopuncture)  Number of treatment: /day /week □ Other ______________________________  (For example, if you treat twice per day, three times per week, indicate this as 2 /day 3 /week)  Treatment period: □ less than 7 days □ more than 7 days □ more than 15 days □ more than 1 month □ more than 2 months □ more than 3 months  □ Others ______________________________ |
| --- |

14. What is the treatment method used for patients who have symptoms ranked number 3 in Question 11? (You should select the most frequently used method.)

□ Herbal medicine (Internal medicine) □ Herbal medicine (External) /< Go to point 14-1 > □ Acupuncture ((If you did not choose acupuncture or pharmacopuncture, the method should be written down in ‘other’) < Go to point 14-2 > □ Cupping □ Moxibustion □ Chuna □ Pharmacopuncture □Other __________________ / < Go to point 14-3 >

| 14-1. If your treatment method is herbal medicine (Internal medicine/External)  Name of drugs: ____________________________________________  Formulation: □ Decoction □ Pills □ Powders □ Others ______________________________  Medical insurance cover: □ Benefit □ Non-benefit □ Others ______________________________  Dosage per day: □ one time per day □ two times per day □ three times per day □ Other ______________________________  Duration: □ less than 3 days □ more than 3 days □ more than 5 days □ more than 7 days □ more than 10 days □ more than 15 days □ more than 30 days □ Others ______________________________ |
| --- |

| 14-2. If your treatment method is acupuncture  Acupoints (3−4 chief acupoints): ____________ ____________ ____________ ____________ ____________  Number of treatment: /day /week □ Others ______________________________  (For example, if you treat twice per day, three times per week, indicate this as 2 /day 3 /week)  Treatment period: □ less than 7 days □ more than 7 days □ more than 15 days □ more than 1 month □ more than 2 months □ more than 3 months  □ Others ______________________________ |
| --- |

| 14-3 If your treatment method is not acupuncture nor herbal medicine (cupping, moxibustion, Chuna, pharmacopuncture)  Number of treatment: /day /week □ Other ______________________________  (For example, if you treat twice per day, three times per week, indicate this as 2 /day 3 /week)  Treatment period: □ less than 7 days □ more than 7 days □ more than 15 days □ more than 1 month □ more than 2 months □ more than 3 months  □ Others ______________________________ |
| --- |

15. Consequently, if you have any opinion or inquiry for integrative medicine research, you can freely write down your opinion.

|  |
| --- |
